# Supplementary material for: Causal Relationship between Aging and Anorexia Nervosa: A White-Matter-Microstructure-Mediated Mendelian Randomization Analysis
Source: Biomedicines. 2024 Aug 16;12(8):1874. doi: 10.3390/biomedicines12081874 (PMC11351342; doi:10.3390/biomedicines12081874)
Supplement: Supplementary file 1 [file biomedicines-12-01874-s001.zip › Supplementary Figure/Figure S8/Leaveoneout plot of MR analysis for TL to ubm-b-1463.pdf]

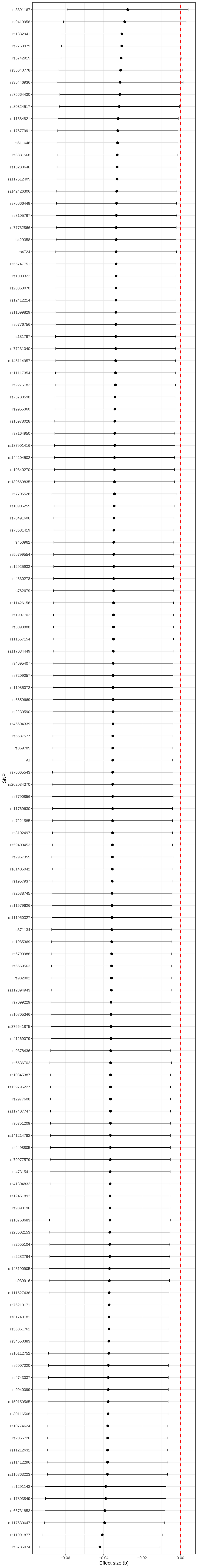
rs3891167
rs9419958
rs1332941
rs2763979
rs5742915
rs35640778
rs35446936
rs75664430
rs80324517
rs11584821
rs17677991
rs611646
rs6881568
rs13230646
rs117512405
rs142426306
rs76666449
rs8105767
rs77732866
rs429358
rs4724
rs55747751
rs1003322
rs28363070
rs12412214
rs11699829
rs6776756
rs131797
rs77231040
rs145114957
rs111117354
rs2276182
rs73730598
rs9955360
rs16978028
rs7164950
rs137901416
rs144204502
rs10840270
rs139669835
rs7705526
rs10905255
rs78491606
rs73581419
rs450962
rs56799554
rs12925933
rs4530278
rs762679
rs11426156
rs1907702
rs3093888
rs11557154
rs117034449
rs4695407
rs7209057
rs11085072
rs6659669
rs2230590
rs45604339
rs6587577
rs869785
All
rs76065543
rs202034370
rs7790856
rs11769630
rs7221585
rs8102497
rs59409453
rs2967355
rs61405042
rs1957937
rs2538745
rs11579626
rs111950327
rs871134
rs1985369
rs6790988
rs6669563
rs932002
rs112394943
rs7099229
rs10805346
rs376641875
rs41269079
rs9878436
rs6536702
rs10845387
rs139795227
rs2977608
rs117407747
rs6751209
rs141214782
rs4498805
rs79977579
rs4731541
rs41304832
rs12451892
rs9398196
rs10768683
rs28502153
rs2555104
rs2282784
rs143190905
rs939916
rs111527438
rs76219171
rs61748181
rs56061761
rs34550383
rs10112752
rs6007020
rs4743037
rs9940099
rs150150565
rs80116508
rs10774624
rs2056726
rs11212631
rs11412296
rs116863223
rs1291143
rs17803849
rs66731853
rs117630647
rs11991877
rs3785074
SNP
-0.06
-0.04
-0.02
0.00
Effect size (b)
